# Vaccine-Preventable Disease Control in the WHO African Region After the COVID-19 Public Health Emergency of International Concern: Implications for Recovery, Resilience, and System Transformation

**DOI:** 10.3390/vaccines14050386

**Published:** 2026-04-26

**Authors:** Charles S. Wiysonge, Abdu A. Adamu, Ado M. Bwaka, Constance N. Wiysonge, Johnson M. Ticha, Reggis Katsande, Andre A. Bita Fouda, Nosheen Safdar, Aschalew Teka Bekele, Chinwe Iwu-Jaja, Blaise Bathondoli, Sidy Ndiaye, Adidja Amani, Maurice Demanou, Samafilan Ainan, Miluka P. Gunaratna, Awa Diop, Yue Han, Anfumbom Kfutwah, Renias Mukaro, Reena H. Doshi, Charles O. Lukoya, Kwasi Nyarko, Jason M. Mwenda, Balcha G. Masresha

**Affiliations:** 1 Cochrane South Africa, South African Medical Research Council, Parrow Valley, Cape Town 7500, South Africa; 2 Department of Global Health, Faculty of Medicine and Health Sciences, Stellenbosch University, Tygerberg, Cape Town 7505, South Africa; 3 Independent Researcher, Kano 700104, Kano State, Nigeria; abdu.adamu@gmail.com; 4 Vaccine-Preventable Diseases Program, World Health Organization Regional Office for Africa, Djoue, Brazzaville P.O. Box 06, Congo; bwakaa@who.int (A.M.B.); tichaj@who.int (J.M.T.); katsander@who.int (R.K.); abita@who.int (A.A.B.F.); safdarn@who.int (N.S.); tekabekelea@who.int (A.T.B.); bbathondoli@gmail.com (B.B.); ndiayes@who.int (S.N.); amaniad@who.int (A.A.); demanou811bis2@gmail.com (M.D.); gunaratnam@who.int (M.P.G.); diopaw@who.int (A.D.); hany@who.int (Y.H.); mukaror@who.int (R.M.); okotc@who.int (C.O.L.); nyarkok@who.int (K.N.); masreshab@who.int (B.G.M.); 5 Department of Mathematical Sciences, Faculty of Applied Sciences, Cape Peninsula University of Technology, Bellville, Cape Town 7535, South Africa; wiysongec@cput.ac.za; 6 Graduate School of Business, Faculty of Commerce, University of Cape Town, Cape Town 7701, South Africa; chinwelolo@gmail.com; 7 Centre for Tropical Diseases and Health, Catholic University of Bukavu, Bukavu P.O. Box 285, Democratic Republic of the Congo; 8 Polio Eradication Program, World Health Organization, Regional Office for Africa, Djoue, Brazzaville P.O. Box 06, Congo; samafilan@gmail.com (S.A.); kfutwaha@who.int (A.K.); 9 Emergency Preparedness and Response Program, World Health Organization, Regional Office for Africa, Brazzaville P.O. Box 06, Congo; rdoshi@who.int; 10 Diarrheal Pathogens Research Unit, Department of Virology, Sefako Makgatho Health Sciences University, Pretoria 0208, South Africa; jason.mwenda23@gmail.com

**Keywords:** vaccine-preventable diseases, immunization recovery, COVID-19, public health emergency of international concern, measles, meningitis, yellow fever, malaria, disease elimination, Africa, health systems

## Abstract

Background: The end of the COVID-19 public health emergency of international concern (PHEIC) in May 2023 marked a transition from disruption to recovery and rebuilding of health systems. The WHO African Region entered this period with declining routine immunization coverage, widening inequities, and fragile surveillance systems. We conducted a critical narrative synthesis of post-PHEIC recovery and the transformation of immunization systems in the region from 2023 to 2025. Methods: We thematically analyzed publicly available data from the WHO and other sources using a systems-oriented framework covering immunization coverage, equity, vaccine introductions, disease control, governance, financing, and data systems. Results: Regional coverage for most antigens was restored to 2019 pre-pandemic levels by 2024, e.g., three doses of diphtheria-tetanus-pertussis-containing vaccines at 76%. However, progress remains insufficient to meet the Immunization Agenda 2030 (IA2030) target of 90% coverage. In addition, there were 6.7 million zero-dose children in the 2024 birth cohort (6.3% higher than the 6.3 million in 2019), concentrated in a few countries. The IA2030 target is a 50% reduction in the number of zero-dose children by 2030, compared to 2019. Recovery initiatives have restored services, while accelerated introductions (e.g., malaria vaccines introduced in 20 new countries in 2024–2025) signal renewed system momentum. Yet, progress has plateaued at pre-pandemic levels, reflecting structural constraints rather than sustained transformation. Concurrently, recurrent outbreaks of measles, yellow fever, and other vaccine-preventable diseases highlight persistent immunity gaps and surveillance limitations. Structural constraints (including financing fragility, subnational inequities, and system fragmentation) continue to limit sustained progress. Conclusion: This study offers important insights that can inform immunization policymaking in the WHO African Region and beyond. Current post-PHEIC trends reflect recovery without transformation. Achieving IA2030 targets will require a shift from broad coverage expansion to precision delivery approaches that prioritize zero-dose and underserved populations. Immunization must be positioned as a central pillar of primary health care and health security systems.

## 1. Introduction

Prior to the coronavirus disease 2019 (COVID-19) pandemic, the World Health Organization (WHO) African Region had achieved steady (though uneven) progress in expanding immunization coverage and reducing morbidity and mortality from vaccine-preventable diseases (VPDs) [1,2]. These gains were driven by the expansion of routine immunization services, the introduction of new vaccines, and periodic supplementary immunization activities (SIAs). However, underlying vulnerabilities persisted. Many countries entered the pandemic with stagnant coverage levels, high dropout rates between vaccine doses, substantial subnational inequities, and fragile surveillance systems insufficiently resilient to systemic shocks [3]. This plateau in performance marks a transition point for immunization systems in Africa; from expansion to precision delivery, where further gains depend on addressing structural constraints and subnational inequities.

The COVID-19 pandemic triggered the most severe disruption to routine immunization services in decades [1]. Lockdowns, movement restrictions, diversion of health personnel, supply chain interruptions, and reduced healthcare utilization contributed to sharp declines in vaccination coverage and delays in outbreak detection and response [4,5]. Between 2020 and 2022, more than 28 million children in the WHO African Region missed the first dose of diphtheria-tetanus-pertussis containing vaccines (DTP1), the conventional proxy indicator for zero-dose status [6]. These disruptions led to the rapid accumulation of susceptible populations and heightened outbreak risk across multiple VPDs.

The declaration in May 2023 of the end of the COVID-19 public health emergency of international concern (PHEIC) marked a transition from emergency response to recovery and system rebuilding [7]. In the WHO African Region, the post-PHEIC years have been characterized not by a simple return to pre-pandemic norms, but by a deliberate effort to restore, adapt, and reposition immunization programs amid persistent fragility, competing crises, and fiscal constraints; offering important lessons for the next phase of the Immunization Agenda 2030 (IA2030) [8].

Despite a growing body of literature on COVID-19-related disruptions to immunization, few analyses have examined the post-PHEIC recovery phase through a systems lens that integrates coverage, equity, governance, financing, and implementation dynamics in the WHO African Region. Existing studies largely focus on pandemic-period disruptions [1,4] or single-program dimensions [2], with limited synthesis of how recovery efforts interact across system components.

This review, therefore, provides a post-PHEIC systems-level synthesis, offering policy-relevant insights into how immunization programs are recovering, transforming, and adapting in the WHO African Region.

## 2. Methods

### 2.1. Data Sources and Selection

We conducted a critical narrative synthesis of secondary data and policy-relevant evidence on post-PHEIC immunization recovery in the WHO African Region. Data were drawn from publicly available sources, including:

- WHO program data and reports [9,10,11,12,13,14,15,16];
- WHO dashboards and surveillance reports [17,18,19,20,21,22,23,24,25,26,27,28,29,30,31,32];
- Policy documents [33,34,35,36,37,38,39,40,41,42,43,44,45,46,47];
- IA2030 Scorecard [48];
- WHO-United Nations Children Fund (UNICEF) Estimates of National Immunization Coverage (WUENIC) [6];
- Peer-reviewed publications [49,50,51,52,53,54,55,56,57,58,59,60,61,62,63,64,65,66,67,68,69].

### 2.2. Inclusion Criteria

We included data sources if they:

- Reported on immunization coverage, vaccine-preventable diseases, or system performance;
- Covered the WHO African Region;
- Were published or updated between 2023 and early 2026;
- Provided quantitative or policy-relevant insights.

### 2.3. Exclusion Criteria

We excluded data sources if:

- They were not relevant to post-PHEIC immunization recovery;
- Data were outdated or superseded by newer estimates;
- They lacked credibility or methodological transparency.

### 2.4. Analytical Framework

We synthesized data using a systems-oriented thematic framework, structured around:

- Coverage and equity;
- Vaccine introductions;
- Disease control and surveillance;
- Regulatory systems;
- Data systems and implementation research;
- Governance;
- Financing and sustainability.

### 2.5. Analytical Approach

We triangulated data across sources to identify trends, compare trajectories across countries where possible, and interpret system-level interactions. We distinguished recovery (return to baseline) from transformation (structural improvement). Post-PHEIC immunization recovery is conceptualized through a complexity lens, in which coverage, equity, surveillance, governance, financing, and delivery platforms interconnect and interact dynamically [70]. Improvements in one domain (e.g., vaccine introductions) do not automatically translate into outcomes (e.g., an increase in vaccination coverage for traditional antigens) unless supported by complementary strengthening across financing, governance, and delivery systems. This framework underpins the analysis presented in this review and emphasizes the need for integrated interventions that target system-oriented leverage points [70].

### 2.6. Methodological Limitations

This study is not a systematic review and does not include an exhaustive literature search, an assessment of risk of bias in the included studies, or a meta-analysis. Targeted searches were enough for the scope of this review. The results of this study rely on secondary data, which may be subject to reporting delays (e.g., the most recent WUENIC and IA2030 scorecard data are for 2024), heterogeneity in data quality, and limited subnational granularity (e.g., WUENIC data are only available at the national level).

## 3. Routine Immunization Coverage and Equity

### 3.1. Recovery at Scale

The immediate post-PHEIC period demanded prompt large-scale recovery efforts. In April 2023, the Big Catch-Up initiative reframed recovery as both a coverage restoration effort and a broader system-strengthening agenda [71]. This initiative (led by WHO; UNICEF; Gavi, the Vaccine Alliance [Gavi]; and the Gates Foundation) aimed to support countries in (a) vaccinating children who missed vaccine doses during the COVID-19 pandemic, (b) restoring coverage to at least 2019 levels, and (c) strengthening primary health care (PHC)-based immunization systems to reach zero-dose children in line with goals of the IA2030 and Gavi’s fifth five-year strategy [72]. By the end of 2025, all pre-selected 24 priority countries in the WHO African Region had initiated Big Catch-Up implementation, vaccinating more than eight million previously zero-dose children and substantially larger numbers of previously under-vaccinated children [18]. This shift signaled the recognition that pandemic-related setbacks could not be addressed through isolated campaigns alone but required integration of catch-up vaccination into routine service delivery, microplanning, and forecasting processes.

### 3.2. Coverage Trends and Zero-Dose Dynamics

Demographic growth has intensified programmatic demands [6]. The number of surviving infants in the region increased by approximately 7%, from 36.6 million in 2019 to 39.2 million in 2024. Against this expanding denominator, regional immunization coverage for most antigens returned to pre-pandemic levels by 2024 but did not substantially exceed them.

DTP1 coverage declined by 3.6% from 83% in 2019 to 80% in 2022, before recovering by 3.8% to 83% in 2024 [6]. Similarly, DTP3 coverage fell by 5.3% from 76% in 2019 to 72% in 2022 and rebounded by 5.6% to 76% in 2024. While these recoveries may demonstrate resilience, they also reveal a structural plateau in coverage. The region has largely returned to 2019 pre-pandemic baselines rather than progressing toward IA2030 targets of 90% coverage for all vaccine doses [8]. This plateau suggests that further progress will require addressing systemic delivery constraints rather than relying solely on incremental expansion of existing approaches.

The annual absolute number of children receiving DTP1 increased by 6.9% from 30.4 million in 2019 to 32.5 million in 2024, reflecting both demographic growth and service restoration [6]. However, the number of zero-dose children in each annual birth cohort rose sharply by 19% during the pandemic (from 6.3 million in 2019 to 7.5 million in 2022) before declining by 10.7% to 6.7 million in 2024. Despite this improvement, the region has not fully reversed pandemic-era setbacks.

Zero-dose children remain highly concentrated geographically [6]. In 2024, ten countries accounted for approximately 80% of the region’s zero-dose burden. Nigeria alone accounted for an estimated 2.1 million zero-dose children (31%), followed by the Democratic Republic of the Congo at 772,000 zero-dose children (11%). This concentration underscores the need for targeted country-specific strategies, with subnational tailoring, rather than uniform regional approaches.

Country-level performance remains heterogeneous [6]. In 2024, DTP3 coverage ranged from 42% in the Central African Republic to 90–94% in thirteen countries, to ≥95% in four countries, including Rwanda, with the highest DTP3 coverage at 98%. These disparities highlight both the feasibility of high performance and the persistent structural inequities limiting progress elsewhere.

While regional coverage for most antigens was restored to 2019 levels by 2024, the absence of an upward trajectory beyond pre-pandemic levels indicates structural stagnation rather than sustained recovery. Countries achieving ≥90% coverage demonstrate feasibility, suggesting that constraints in lower-performing settings are not technical but systemic, linked to governance, financing, and subnational delivery capacity.

### 3.3. Institutionalizing Catch-Up and Addressing Demand

A notable policy evolution during the post-PHEIC period has been the normalization of catch-up vaccination beyond infancy [33]. More than 20 countries revised policies to vaccinate children up to five years of age, embedding catch-up as a routine function rather than a time-bound campaign response [18]. This policy evolution, strongly endorsed by regional technical advice, reflects a more resilient approach to inevitable disruptions; whether from pandemics, conflict, supply constraints, or climate shocks [33].

Recovery strategies have increasingly recognized that supply restoration alone is insufficient. Several countries have expanded the use of behavioral and social data to tailor communication strategies, address vaccine hesitancy, rebuild trust following COVID-19 disruptions, and mitigate indirect access costs faced by caregivers. As of mid-2025, at least 20 countries had conducted assessments of behavioral and social drivers to guide demand-generation strategies, and 26 had vaccine-related legislation reinforcing immunization as a core public health function [17].

### 3.4. Persistent Equity Gaps

Despite measurable recovery, equity gaps remain pronounced. Zero-dose and under-vaccinated children continue to be disproportionately concentrated in remote rural areas, informal urban settlements, conflict-affected and fragile settings, border regions, and among other marginalized populations [73]. Although comprehensive subnational data remain limited, this clustering indicates that national averages mask substantial intra-country inequities, reinforcing the need for embedded implementation research, targeted microplanning, and context-specific strategies.

High dropout between DTP1 and DTP3 persists in several settings, indicating challenges in sustaining contact with the health system beyond the first vaccination visit. Overall, in the WHO African Region, the number of infants who started but did not complete the full series of three DTP doses each year increased from 2.5 million in 2019 to 3.1 million in 2022 before decreasing slightly to 2.8 million in 2024; with wide variation across and within countries [6]. These patterns reinforce the need to shift from population-wide strategies to precision delivery approaches tailored to high-risk and underserved populations.

While regional recovery demonstrates that progress is possible even in constrained environments, the evidence suggests that restoration to pre-pandemic coverage levels is insufficient. Achieving IA2030 targets will require deliberate strategies to reduce dropout rates, strengthen the second-year-of-life platform, institutionalize catch-up mechanisms, enhance subnational microplanning, and integrate immunization more deeply into PHC systems. The post-PHEIC period therefore represents not merely a phase of recovery but a strategic opportunity to redesign immunization delivery for resilience and equity.

## 4. New Vaccine Introductions

One of the clearest markers of renewed health system momentum in the post-PHEIC period in the WHO African Region has been accelerated national introductions of new and under-utilized vaccines. In 2023, there were 11 new vaccine introductions, and in 2024, there were 28 [48]. In particular, there was accelerated progress in the introduction of malaria vaccines (no new country in 2023 but 13 new countries in 2024), the second dose of the inactivated polio vaccine (IPV2: 7 in 2023 and 6 in 2024), the second dose of measles-containing vaccines (MCV2: 1 each in 2023 and 2024), and human papillomavirus (HPV) vaccines (3 in 2023 and 1 in 2024) [45]. These trends align with IA2030’s emphasis on life-course vaccination and integrated service delivery, but they also sharpen a central policy challenge: ensuring that the pace of vaccine introduction is matched by the strength and equity of delivery systems.

Beyond their direct disease impact, these introductions have functioned as practical system stress tests. The introductions require countries to strengthen regulatory systems, delivery platforms, cold chain capacity, data systems, safety surveillance, and community engagement, often under tight timelines and within constrained fiscal space. In several settings, new vaccine introductions have therefore served as catalysts for broader PHC integration, life-course immunization approaches, and renewed attention to reaching missed and under-vaccinated populations [20,22,49]. At the same time, uneven readiness, persistent financing gaps, and variable demand dynamics mean that rapid introduction does not automatically translate into high, sustained coverage, reinforcing the importance of deliberate implementation design [20,22].

### 4.1. Malaria Vaccine Introduction

The malaria vaccine rollout represents a landmark shift from pilot implementation to broader programmatic scale in Africa. Building on the pilot Malaria Vaccine Implementation Program experience in Ghana, Kenya, and Malawi, a rapid expansion phase has followed, supported by coordinated technical assistance and partner alignment [53]. The WHO-led multi-partner platform (Accelerating Malaria Vaccine Introduction and Rollout in Africa) launched in January 2024 has helped countries navigate planning, training, logistics, demand generation, and monitoring, while strengthening coordination across national and regional stakeholders [22,54].

By October 2025, there were 23 countries that had introduced malaria vaccines into their national immunization schedules, reflecting a rapid expansion from three pilot countries in December 2023 [19,53]. Scale-up has also been accompanied by efforts to strengthen performance intelligence, including digital dashboards and real-time monitoring tools to support rapid corrective actions during rollout [19,22,54]. However, sustaining malaria vaccine delivery will require reliable integration into routine child health platforms, stable financing for operations and supervision, and careful management of community expectations, particularly in settings with high malaria burden and concurrent health system constraints.

### 4.2. HPV Vaccine Introduction

HPV vaccination has emerged as a cornerstone of life-course immunization in the WHO African Region, anchored in the cervical cancer elimination agenda. Despite progress, coverage remains below the levels required to meet the global 90–70–90 targets, and the post-PHEIC period has highlighted that demand-side barriers and delivery complexity can constrain performance even when political commitment exists [55]. Nevertheless, the region has continued to expand HPV introductions and refine delivery strategies, with many countries transitioning to WHO-recommended single-dose schedules, which simplifies implementation and improves feasibility within constrained systems [20,56].

By the end of 2025, a total of 35 countries in the region had introduced HPV vaccination into their routine immunization schedules (up from 25 in 2022), and 47% of girls had received at least one dose by age 15 years in the region [20]. Several countries have achieved high coverage, demonstrating feasibility when school-based strategies are well planned, communities are engaged early, and health worker capacity is strengthened [55]. At the same time, implementation has faced supply and logistics bottlenecks in some settings; vaccine hesitancy and misinformation, including anti-HPV narratives amplified in the post-COVID information environment; and uneven healthcare worker confidence and training on HPV vaccine benefits and eligibility [55,56]. The post-PHEIC period is therefore strategically important, not only for introductions, but also for embedding HPV vaccination within routine platforms in ways that sustain coverage, build trust, and protect equity.

One of the most consequential policy shifts shaping this period has been the move to single-dose HPV schedules [33]. The latter were first recommended by the Strategic Advisory Group of Experts on Immunization in April 2022, formalized in the updated WHO position paper on HPV vaccines in December 2022 [74], and subsequently endorsed for the WHO African Region by the Regional Immunization Technical Advisory Group (RITAG) in November 2023 [33]. A total of 29 countries in the region had transitioned from two-to-three dose schedules to one-dose schedules by the end of 2025 [20]. The transition to single-dose HPV schedules represents one of the most operationally significant policy shifts in the region’s immunization program in recent years. For high-burden, resource-constrained settings, single-dose schedules reduce the logistical complexity of follow-up and extend the reach to adolescent girls who may have limited repeat contact with health services. Evidence from early adopters in the region suggests that single-dose programs, when paired with strong school-health linkages and community mobilization, can achieve high coverage levels [20,55]. However, equity considerations must remain central: out-of-school girls, those in humanitarian settings, and adolescents in hard-to-reach communities remain systematically under-reached by school-based delivery, requiring deliberate complementary strategies through community and health facility platforms.

Looking ahead, the integration of HPV vaccination into broader life-course health frameworks (linking adolescent immunization contacts with sexual and reproductive health, nutrition, and mental health services) offers both a programmatic and political opportunity to elevate the visibility of adolescent health on national agendas. Several countries in the region are piloting integrated adolescent health platforms that bundle HPV vaccination with other interventions, an approach that could improve cost-efficiency and strengthen the case for domestic financing [55,56]. Institutionalizing these platforms, rather than treating HPV delivery as a vertical program, will be essential to sustaining coverage gains beyond donor-supported introduction phases.

### 4.3. Introduction of Under-Utilized Vaccines

Beyond malaria and HPV, countries have continued to expand the uptake of multiple under-utilized vaccines that are central to accelerated VPD control and epidemic preparedness. These include the continued rollout of IPV2 and meningococcal conjugate vaccines, and broadening the second-year-of-life platform through additional vaccine doses such as MCV2 and rubella-containing vaccines [48]. Collectively, these introductions support a shift away from a narrow infant-only immunization paradigm toward a more resilient life-course approach to immunization.

However, the region’s experience also underscores that introduction is not the endpoint. The operational bottlenecks that limit routine coverage (including health workforce shortages, weak microplanning for underserved populations, stock management challenges, and fragmented data systems) can also limit the impact of newly introduced vaccines [54,55]. Where countries have been most successful, introductions have been paired with deliberate system strengthening, including stronger cold chain planning, integrated supportive supervision, catch-up strategies for missed cohorts, and targeted community engagement to build trust and reduce drop-out.

### 4.4. Vaccine Introductions as a Platform for System Transformation

The post-PHEIC acceleration of vaccine introductions offers a time-limited opportunity to enable system improvements that extend beyond any single antigen. When designed intentionally, introductions can strengthen PHC platforms, improve data visibility, and build delivery capacity for reaching underserved populations, thus directly advancing equity goals. Conversely, when introductions outpace readiness, they risk becoming episodic events that add workload without improving routine performance, potentially widening inequities if better-served districts adopt and sustain coverage faster than hard-to-reach ones.

A key lesson is therefore that the region’s vaccine introduction agenda must be paired with (a) predictable financing for operational delivery, (b) integration into routine contacts beyond infancy, (c) strong safety surveillance and risk communication, and (d) performance management systems that identify and address subnational gaps rapidly. Framed this way, new vaccine introductions are not only a measure of technical progress, but also a practical pathway for strengthening resilient immunization systems capable of sustaining disease control gains in the post-PHEIC era.

## 5. Accelerated Disease Control

The post-PHEIC period in the WHO African Region has been characterized by a predictable but serious epidemiological aftershock. The accumulation of susceptible cohorts has translated into intensified VPD outbreaks, including (a) the occurrence of large and disruptive measles outbreaks in half of the countries in the region, (b) multiple countries documenting circulating vaccine-derived polio viruses (cVDPVs), and (c) the unusual occurrence of diphtheria outbreaks in at least nine countries [9,10,11,12,13,14,36,48,57,58,59]. These events are not simply episodic shocks; they are symptoms of persistent immunity gaps and a direct test of routine immunization performance, the quality of SIAs, and the timeliness of outbreak response. In this context, immunization functions simultaneously as a child survival intervention and as a core component of health security.

Across the region, preparedness and response efforts have increasingly emphasized integrated disease surveillance, including expanded environmental surveillance for polioviruses [57,58,59,60]. While the speed and quality of outbreak detection and response remain uneven, several countries have demonstrated more coordinated actions than in the pre-pandemic period, drawing explicitly on operational lessons from COVID-19 [33]. This section summarizes progress and remaining constraints in measles and rubella elimination, elimination of yellow fever and meningitis epidemics, maternal and neonatal tetanus elimination, and polio eradication and transition. It highlights the central insight of the post-PHEIC era: outbreak control accelerates when routine systems, second-year-of-life platform, and surveillance are strengthened together, rather than treated as parallel workstreams.

### 5.1. Measles and Rubella Elimination

From 2000 to 2024, the WHO African Region has achieved major reductions in measles mortality, with an estimated 91% decline in annual deaths (from 391,690 to 33,639) [13]. However, progress with measles elimination in the region remains structurally uneven [9,49,50]. Cabo Verde, Mauritius, and Seychelles were verified for measles and rubella elimination in November 2025, demonstrating that elimination is feasible in the region when high routine two-dose measles-containing vaccine (MCV) coverage and sensitive surveillance are sustained [9,35]. However, no large-population country has yet reached verification, and ongoing measles transmission in the region continues to reflect the accumulation of susceptible children in high-burden settings.

Rubella-containing vaccine (RCV) introduction has expanded rapidly in the region, with 35 of 47 countries incorporating RCV into routine immunization schedules by 2024 [50,51]. Surveillance data analyses from early-adopting countries show an average 76% reduction in reported rubella cases by 2024 [51]. However, persistent rubella transmission in countries yet to introduce the vaccine highlights the importance of maintaining high population immunity and sensitive surveillance to achieve regional measles-rubella elimination.

Surveillance data illustrate the persistence of measles transmission across the region [9,10,11,12,13]. In 2024, the region reported 147,564 suspected measles cases through case-based surveillance, with 77,698 confirmed; corresponding to a confirmed measles incidence of 58.8 per million population. These figures signal that routine immunization coverage is not yet high enough to reliably interrupt measles transmission in the region.

The central bottleneck remains low routine two-dose MCV coverage [6]. Coverage with the first dose of MCV (MCV1) decreased sharply from 71% in 2019 to 67% in 2022, before recovering to 71% in 2024. Coverage with MCV2 increased from 32% in 2019 through 43% in 2022 to 55% in 2024, driven by new introductions; 44 of the 47 countries in the region had introduced MCV2 by the end of 2024. However, there remains a large gap between MCV1 and MCV2 in many countries. This pattern points to a weak second year of life platform, where drop-out and missed opportunities for vaccination remain common. Generally, countries that have integrated additional antigens into the second year of life contacts tend to show higher MCV2 uptake and lower dropout, implying that service integration can strengthen measles performance beyond measles-specific programming.

In response to widening immunity gaps, countries have intensified preventive mass vaccination campaigns (PMVCs) and outbreak response campaigns [9,10,11,12,13]. In 2023–2024, a total of 24 countries implemented 28 preventive and six large-scale outbreak-response measles/measles-rubella SIAs, collectively vaccinating more than 164 million children [10]. In addition, in 2025, more than 74 million children in the region were protected against measles through SIAs in 16 countries [9].

Operational refinements, such as shifting from 10-dose to 5-dose MCV vials, appear to have reduced missed opportunities for vaccination by increasing session flexibility and improving the confidence of healthcare workers to open vials beyond high-attendance days [52]. However, the region’s measles-rubella elimination efforts remain constrained by weak surveillance in many countries. In 2024, only 27of 47 countries met annual targets for the two principal measles surveillance indicators, constraining timely detection, confirmation, and response [10].

The attainment of sustainable measles-rubella elimination in the WHO African Region will require robust routine vaccination delivery systems that reliably achieve high two-dose coverage, alongside consistently high-quality and timely SIAs targeted to known immunity gaps, and surveillance systems capable of rapidly detecting and confirming transmission at subnational levels; particularly in high-burden, conflict-affected, and highly mobile contexts.

### 5.2. Elimination of Yellow Fever Epidemics

Yellow fever is a persistent public health challenge in Africa, driven by immunity gaps and increasingly by ecological and demographic change [23,24,25,26,27,28,29,30,31]. Following yellow fever outbreaks in Angola and the Democratic Republic of the Congo with international spread in 2016, the global strategy to Eliminate Yellow Fever Epidemics (EYE) 2017–2026 framed yellow fever control around three linked goals: protecting at-risk populations, preventing international spread, and rapidly containing outbreaks [24]. The African regional framework to operationalize EYE was adopted in 2017 and set clear deliverables by end-strategy in December 2026. The latter includes (a) introduction of yellow fever vaccination into routine immunization schedules of all 27 countries at highest risk of yellow fever outbreaks; (b) completion of PMVCs in all 27 priority countries; (c) expanded national diagnostic capacity; and (d) functional regional reference laboratory capacity [25].

Routine vaccine introduction has advanced, but with important caveats for risk stratification and subnational equity [25,26,27]. By 2025, twenty-five of twenty-seven high-risk countries in the region had introduced yellow fever vaccination into routine schedules, reflecting substantial alignment with the EYE strategy. However, implementation has not been uniform. In Kenya, routine yellow fever vaccination was introduced only in subnational geographies previously classified as high-risk but subsequent outbreaks have occurred in areas previously classified as low-risk, illustrating the limitations of static risk classifications and the need for adaptive risk mapping. Ethiopia and South Sudan had not introduced routine yellow fever vaccination by the close of 2025, leaving significant immunity gaps in populations already vulnerable to epidemic spread. These variations highlight both the progress achieved and the persistent challenges in ensuring equitable and comprehensive protection against yellow fever outbreaks in the WHO African Region.

Since the inception of EYE in 2017, the scale-up of yellow fever SIAs has been substantial. By the end of 2025, an estimated 444 million individuals had been immunized through SIAs, reflecting one of the largest coordinated mass vaccination efforts in the region [10,25,27]. In 2024 alone, 54.7 million people received yellow fever vaccine doses, of which 48.9 million were vaccinated during PMVCs and 5.8 million through reactive SIAs [25,27]. In addition, about 12.3 million were protected in 2025 (10.9 million through PMVCs and 1.4 million through reactive SIAs) [10]. PMVC implementation is progressing, but remains incomplete [10,25,27]. By the end of 2025, twenty countries had completed PMVCs, strengthening population-level immunity against yellow fever. Two high-risk countries (the Democratic Republic of the Congo and Niger) remain in the midst of extended implementation, with ongoing multi-year PMVCs to ensure comprehensive coverage. In contrast, one country (Ethiopia) is still in the planning phase, and four countries (Equatorial Guinea, Gabon, Kenya, and South Sudan) have yet to initiate PMVCs, leaving substantial immunity gaps in populations vulnerable to epidemic spread. This heterogeneity underscores the need for sustained political commitment, predictable financing, and adaptive operational strategies to ensure that PMVCs are implemented comprehensively across all high-risk settings.

The substantial immunity gaps perpetuate the risk of yellow fever outbreaks. During the period 2024–2025, seventeen countries in the region reported 248 probable or confirmed yellow fever cases, with the highest-burden countries being Ghana (47 cases), Uganda (*n* = 41), Nigeria (*n* = 37), Burkina Faso (*n* = 24), Cameroon (*n* = 21), and Chad (*n* = 17) [27]. In addition, five deaths were documented, including two in Cameroon, two in Uganda, and one in The Gambia. This corresponds to a case fatality rate of 3.0% for both confirmed and probable cases and 3.3% for confirmed cases. Therefore, the risk of yellow fever outbreaks remains real where population immunity is uneven, vector ecology is favorable for transmission, and surveillance is fragmented [27].

Surveillance and laboratory capacity for yellow fever diagnosis are improving in the WHO African Region but remain a limiting factor for confident risk assessment and rapid response to yellow fever outbreaks [25]. By the end of 2025, a total of 29 national laboratories in 24 high-risk countries had established serological diagnostic capacity for yellow fever. However, only 16 laboratories across 13 countries had achieved full accreditation by WHO, and only 16 countries had implemented yellow fever routine molecular testing, as evidenced by molecular proficiency assessments conducted by WHO in 2025. This remains a limitation to the reliability and comparability of diagnostic outputs across the region. At the regional level, confirmation capacity is supported by three WHO-accredited reference laboratories: the Centre Pasteur du Cameroun, the Institut Pasteur de Dakar, and the Uganda Virus Research Institute [25]. These institutions provide critical support for case confirmation, quality assurance, and training. Despite these advances, the uneven distribution of accredited laboratories and persistent surveillance gaps constrain timely risk assessment and outbreak response, underscoring the need for sustained investment in laboratory systems and integrated surveillance networks.

Despite substantial progress in vaccination and surveillance, several emerging challenges threaten yellow fever control in the WHO African Region [23,24,25,26,27]. The urban proliferation of *Aedes aegypti* mosquitoes and daytime biting patterns have heightened the risk of explosive outbreaks in densely populated cities, where transmission dynamics are amplified by high population mobility and limited vector control. In addition, changing ecological and demographic patterns (including rapid urbanization, climate variability, and shifting migration flows) are increasingly outpacing static risk classifications, underscoring the need for adaptive and dynamic risk mapping. Finally, surveillance gaps and limited laboratory accreditation constrain the timeliness and reliability of outbreak detection and response. Together, these factors highlight the necessity of strengthening integrated surveillance systems, expanding laboratory accreditation, and developing flexible preparedness strategies capable of responding to evolving epidemiological landscapes.

### 5.3. Elimination of Epidemic Meningitis

The African Meningitis Belt provides one of the clearest demonstrations of what sustained vaccination can achieve at scale [58,59]. Before 2010, *Neisseria meningitidis* serogroup A caused almost 90% of epidemics. The rollout of meningococcal A conjugate vaccine (MenACV) across 24 of 26 meningitis-belt countries from 2010 to 2024, via PMVCs and routine immunization, resulted in a marked decline in serogroup A epidemics and an epidemiological shift toward other serogroups (C, W, X) and *Streptococcus pneumoniae* [58]. Quantitatively, the MenACV rollout was associated with a two-thirds reduction in cases. Separately, adoption of ceftriaxone as first-line treatment from 2015 reduced case fatality rates by half [59].

The post-PHEIC landscape is therefore not a return of serogroup A, but a shift to multivalent complexity requiring vaccine innovation, responsive outbreak tools, and sustained surveillance. In 2024, Niger and Nigeria (which were experiencing serogroup C and W epidemics) introduced the new pentavalent meningococcal ACWYX conjugate vaccine (Men5CV) in reactive SIAs, vaccinating almost five million people aged 1–29 years and stopping the epidemics [59]. Meningitis risk assessments were also conducted in Burkina Faso, Ghana, Mali, Niger, and Nigeria; and PMVCs using ACWY vaccines were conducted in Cameroon and Togo [59]. In addition, surveillance was strengthened through support to 24 meningitis-belt countries and Angola [59].

However, three constraints threaten accelerated progress in meningitis control [33,59]. Firstly, competing demands from multiple new vaccine introductions can delay the adoption of MenACV or Men5CV in countries still building fiscal and delivery capacity. Secondly, co-financing for Men5CV may be challenging for countries in transition from Gavi support (such as Ghana and Nigeria), risking delayed routine integration even where outbreak risk is high. Thirdly, the recent discontinuation of donor funding for a regional surveillance network in the meningitis belt creates a critical vulnerability. Without stable support for high-quality case-based surveillance at representative sites, decision-making becomes slower and less precise at the moment when multivalent preparedness is most needed [59].

Efforts to eliminate meningitis epidemics in the WHO African Region have moved from a one-vaccine solution to a multivalent preparedness agenda. Sustaining gains will require predictable financing, stronger case-based surveillance and laboratory confirmation, and timely access to next-generation vaccines aligned with the “Defeating Meningitis by 2030” trajectory [33,59].

### 5.4. Maternal and Neonatal Tetanus Elimination

Maternal and neonatal tetanus (MNT) remains a marker of inequity in access to clean delivery and effective immunization services. Despite substantial progress over two decades, MNT persists where health system reach is weakest [60]. As of December 2025, a total of 44 of 47 countries in the WHO African Region had eliminated MNT [60]. The remaining countries are Angola, the Central African Republic, and Nigeria. Nigeria shows subnational progress, with elimination validated in the southeast, southwest, south, and north-central zones.

Progress post-PHEIC includes the validation of MNT elimination in Mali, Guinea, and South Sudan, and the successful conduct of post-validation assessments in Gabon and Côte d’Ivoire, demonstrating maintenance of elimination status [60]. However, the binding constraint for the remaining countries is operational rather than technical; limited resources to conduct PMVCs and reach underserved communities, especially where conflict, weak routine contact with pregnant women, and health workforce constraints intersect.

Completing MNT elimination in the WHO African Region is achievable, but it is a classical last-mile problem, requiring targeted financing and delivery strategies to reach underserved communities with low-skilled birth attendance and weak routine immunization access [60].

### 5.5. Polio Eradication and Transition

Polio eradication in the WHO African Region captures the dual reality of remarkable achievement and unfinished business [33,57]. The region has remained free of indigenous wild poliovirus since August 2020, and paralysis due to cVDPVs has declined by more than 90% from 2022 to 2025 [37,38]. Nevertheless, cVDPV outbreaks continue to occur and remain a persistent risk indicator for immunity gaps and uneven performance in outbreak response and routine immunization coverage.

#### 5.5.1. Epidemiology and Transmission Dynamics

A critical and underappreciated dimension of the post-PHEIC period has been the increasing complexity of the cVDPV landscape in the region. While the total cVDPV case burden has declined substantially since its 2022 peak, outbreaks are now more geographically dispersed with simultaneous transmission across multiple countries. This pattern is often driven by cross-border population movement, insecurity, and persistent pockets of zero-dose and under-vaccinated children, making outbreak response more operationally demanding [57]. The Lake Chad Basin has emerged as a persistent reservoir of cVDPV2 transmission, with Nigeria and Chad contributing disproportionately to both regional and global caseloads [38,39]. In addition, the epidemiological profile has diversified. cVDPV3 re-emerged in Guinea, Cameroon, and Chad during 2024–2025, and in Nigeria in early 2026, while cVDPV1 cases have been reported in Algeria and the Democratic Republic of the Congo [32,40]. These trends underscore persistent vulnerability and continued risks of cross-border transmission in areas with large numbers of zero-dose and under-vaccinated children. Additionally, orphan poliovirus detections across the region, reflecting undetected silent transmission chains, further underscore that case counts alone underestimate the true scale of circulation and the underlying programmatic challenge [61].

#### 5.5.2. Surveillance and Laboratory Systems

Despite these challenges, the surveillance and laboratory platform for polio remains one of the strongest public health assets in the WHO African Region [32,61,62]. Fifteen countries are currently classified as priority due to persistent transmission or high outbreak risk and receive targeted support through the Global Polio Eradication Initiative, while other countries increasingly rely on integrated vaccine-preventable disease surveillance as polio-specific assets transition [32].

The regional laboratory network includes sixteen WHO-accredited polio laboratories across fifteen countries, supporting both acute flaccid paralysis surveillance and environmental surveillance. By 2025, a total of 46 of the 47 countries in the region were implementing environmental surveillance, substantially strengthening the sensitivity of poliovirus detection and enabling earlier identification of transmission [32,61,62].

#### 5.5.3. Policy Implications and Transition

Post-PHEIC, it has become increasingly clear that sustaining polio eradication gains depends less on vertical polio programming and more on the strength and integration of routine immunization systems, surveillance platforms, and outbreak response capacity. Continued political engagement, integration of polio functions into broader health systems, and accelerated introduction of additional IPV doses are central to preventing resurgence and enabling viable transition.

Polio eradication in the region has therefore evolved into a test of system integration and sustainability. The extensive assets built through the Global Polio Eradication Initiative (including surveillance systems, skilled workforce, laboratory networks, microplanning capacity, data systems, and accountability mechanisms) must be deliberately absorbed into routine immunization and broader vaccine-preventable disease control, rather than lost during transition.

At present, polio transition (the integration of essential polio functions into national systems with sustainable domestic financing) has not yet become an immediate operational reality in the WHO African Region [41]. Interrupting cVDPV2 transmission in persistent reservoir countries remains the primary unfinished task, and any premature or poorly sequenced transition risks undermining outbreak response capacity.

Nevertheless, the transition planning horizon must begin now. The institutional knowledge, operational discipline, and community engagement infrastructure established under the Global Polio Eradication Initiative represent irreplaceable public health assets. Ensuring that transition is managed as a deliberate, phased, and adequately financed process (rather than a passive consequence of declining donor support) should be a central priority of the post-PHEIC immunization policy agenda in the WHO African Region.

## 6. Monitoring Vaccine Effectiveness

Monitoring vaccine effectiveness in real-world conditions is essential to inform immunization policies, particularly in the context of population immunity gaps, emerging pathogen variants, and changing epidemiology. To address the limited availability of vaccine effectiveness evidence from Africa, the WHO Regional Office for Africa and partners established the African Region Monitoring Vaccine Effectiveness (AFRO-MoVE) Network in March 2021 [15]. The network was initially designed to facilitate and coordinate COVID-19 vaccine effectiveness studies across the region, promote the use of standardized study designs to enable pooled regional analyses, and build sustainable capacity among hospitals, laboratories, and research institutions to evaluate vaccines against respiratory pathogens.

AFRO-MoVE supports vaccine effectiveness studies using harmonized methodologies, including prospective cohort studies among priority groups such as healthcare workers and test-negative case–control studies embedded within severe acute respiratory infection surveillance systems. Through technical guidance, training, and a centralized data platform for pooled analyses, the network has brought together more than 200 experts from 22 African countries and over 50 organizations. This coordinated platform strengthens the region’s ability to generate locally relevant evidence on vaccine performance, inform immunization strategies, and enhance preparedness for future epidemics and pandemics [15].

Post-PHEIC, sustaining and expanding vaccine effectiveness monitoring platforms in countries of the WHO African Region will be critical to inform vaccine policy optimization, guide the introduction of new vaccines, and support preparedness for emerging and re-emerging infectious threats. This was demonstrated in recent rotavirus vaccine effectiveness studies in the region [63].

## 7. Governance and Advisory System

Technical progress in immunization is inseparable from governance quality. The post-PHEIC period has reaffirmed that strong immunization systems depend on credible advisory bodies, coordinated partner platforms, and sustained political stewardship [42,75]. In the WHO African Region, governance architecture for immunization has matured considerably over the past decade, particularly through the RITAG, Regional Working Groups on immunization, National Immunization Technical Advisory Groups (NITAGs), and strengthened national coordination mechanisms such as Inter-Agency Coordinating Committees (ICCs). However, variability in functionality and maturity continues to shape program performance [66].

### 7.1. RITAG

At the regional level, the RITAG provides independent technical advice to the WHO Regional Office for Africa, helping harmonize policy recommendations and interpret emerging evidence in the African context. RITAG has played a key role in recommending catch-up vaccination beyond infancy, guiding the measles elimination strategy, advising on HPV schedule transitions, supporting malaria vaccine rollout, and other initiatives [33,34].

Although the Strategic Advisory Group of Experts on Immunization sets global standards [75], its guidance may not fully address Africa-specific epidemiological patterns and health system constraints, highlighting the critical role of the Regional Immunization Technical Advisory Group in adapting recommendations to regional realities. Regional advisory coherence is particularly important in Africa, where epidemiological patterns (e.g., meningitis belt dynamics, yellow fever risk stratification, and high zero-dose concentration) differ from global patterns. RITAG serves as the bridge between global normative guidance and regional operational realities, reinforcing policy consistency while preserving national decision-making authority. Post-PHEIC, RITAG has held two online meetings on emerging issues (including mpox, cholera, and yellow fever outbreak response) and one annual face-to-face meeting, with the first meeting taking place in November 2023 in Brazzaville, Congo [33,34].

### 7.2. NITAGs

NITAGs are multidisciplinary, independent national specialist bodies that provide evidence-informed recommendations to ministries of health on vaccine policy and immunization strategy [42].

As of December 2025, a total of 45 of the 47 countries (representing 99% of the region’s population) have established NITAGs. Thirty-six (94%) meet WHO’s six criteria for functionality, and 18 have maintained functionality for at least five consecutive years [17]. This marks substantial progress compared with a decade earlier and reflects intensified regional coordination since 2019 [17,42]. However, functionality does not equate to maturity. Twenty-eight countries have applied the NITAG Maturity Assessment Tool; of these, 20 achieved overall maturity scores above 50% [66]. Importantly, maturity levels are not strictly correlated with the age of the NITAG, suggesting that institutional design, secretariat support, access to evidence, and political positioning are as important as longevity.

NITAG maturity scores reflect substantive differences in countries’ ability to translate evidence into policy and implementation. Currently available evidence suggests that higher-maturity NITAGs are more likely to generate timely, context-specific recommendations that are integrated into national decision-making, whereas lower-maturity NITAGs often face delays, limited contextualization, and weaker policy uptake [42]. These differences have programmatic implications. Governance effectiveness, therefore, depends not only on the presence of advisory bodies but on their institutional maturity and integration within financing, coordination, and delivery systems; an increasingly critical determinant of immunization performance in the post-PHEIC period.

The post-PHEIC period has increased the strategic relevance of NITAGs. Decisions regarding catch-up vaccination policies, HPV single-dose schedules, malaria vaccine introduction, and Men5CV deployment have required rapid yet evidence-based guidance. Current priority topics on the agenda of NITAGs in the region include vaccine introduction prioritization and sequencing, as well as vaccine portfolio optimization. Where NITAGs are strong, vaccine decisions have been more transparent, context-sensitive, and fiscally realistic. Where advisory processes are weak, delays or politically driven decisions have sometimes complicated implementation [42]. Thus, variability in governance capacity across countries continues to influence the speed and consistency of policy adoption and implementation.

### 7.3. Partner Platforms

ICCs function as coordination hubs linking ministries of health, WHO, UNICEF, Gavi, civil society, and other partners [72]. During the Big Catch-Up period and subsequent recovery efforts, effective ICCs facilitated rapid resource mobilization, synchronized campaign planning, and aligned partner support. Conversely, weak coordination structures contributed to fragmented planning and implementation delays.

The WHO African Region has two Regional Working Groups on Immunization: one for the two subregions of West and Central Africa and the other for the subregion of East and Southern Africa [33]. In the post-PHEIC period, Regional Working Groups have enhanced partner coordination, especially within the context of the Big Catch-Up initiative [72]. During this period, each of the three subregions (i.e., Central Africa, East and Southern Africa, and West Africa) has held annual face-to-face meetings of national immunization managers to strengthen peer learning and accountability among countries at the sub-regional level. In addition, high-level political engagements such as the immunization side-event at the 74th Session of the WHO Regional Committee for Africa in August 2024, have reinforced ministerial commitments and elevated immunization within broader health security and development discourse [43].

However, governance challenges remain. In several countries, NITAG recommendations are not always systematically integrated into national immunization policies and budgeting processes [42]. ICCs may meet irregularly or function primarily during Gavi application cycles rather than as continuous oversight platforms. Surveillance data are not always systematically fed back into policy deliberations. Strengthening governance, therefore, requires institutionalizing regular review cycles, improving transparency of decision-making, and ensuring that technical advice is directly linked to financing and operational planning.

### 7.4. Governance as a Determinant of Resilience

The post-PHEIC experience suggests that governance quality is a determinant of resilience. Countries with functional NITAGs and active ICCs were generally quicker to normalize catch-up vaccination, introduce new vaccines strategically, and respond to outbreaks [72]. Governance structures act as the connective tissue linking evidence, financing, and implementation. Moving forward, governance strengthening should focus on enhancing NITAG maturity and secretariat capacity; institutionalizing integration between NITAG recommendations and national budget processes; strengthening ICC oversight beyond donor compliance functions; promoting transparency and accountability in vaccine decision-making; and aligning immunization governance with broader PHC reform agendas.

In all, technical capacity alone cannot sustain VPD control. Durable progress requires governance systems that are credible, coordinated, and financially anchored; ensuring that evidence-informed policy translates into sustained implementation.

## 8. Strengthening Vaccine Regulatory Systems

Strengthened regulatory systems have been an important, though often under-recognized, component of immunization recovery and innovation in the WHO African Region in the post-PHEIC period. The African Vaccine Regulatory Forum (AVAREF), coordinated by the WHO Regional Office for Africa, has continued to serve as a critical platform for regulatory collaboration, capacity building, strengthening, and harmonization among national regulatory authorities and national ethics committees [64]. Through joint reviews of clinical trial applications, scientific advice to vaccine developers, facilitated product registrations, and support for expedited regulatory pathways, AVAREF has helped streamline and strengthen decision-making processes while maintaining rigorous standards of safety, quality, and efficacy. The AVAREF platform was used in facilitated registrations for malaria and pentavalent meningococcal conjugate vaccines. From 2023 to 2025, AVAREF expanded its role beyond clinical trial oversight to support regulatory preparedness for vaccines for emerging pathogens, facilitate reliance mechanisms among countries, and strengthen alignment between regulatory review and immunization program priorities [16,64].

The value of this regional regulatory collaboration was recently demonstrated during the mpox outbreaks in Africa, when AVAREF coordinated a multi-country joint review of the mpox vaccine dossier following the declaration of mpox as a PHEIC in August 2024 [16]. Fourteen high-risk countries jointly assessed the available safety and effectiveness evidence and completed the scientific review within approximately 20 working days of the declaration of mpox as a PHEIC. This enabled national regulatory authorities to proceed with emergency use authorization for their use in their country’s immunization programs. By September 2025, seventeen African countries had authorized the use of the vaccine, illustrating how regulatory reliance and coordinated review mechanisms can accelerate access to life-saving countermeasures during public health emergencies while preserving national regulatory sovereignty.

Beyond emergency response, AVAREF has increasingly positioned itself as a strategic platform supporting Africa’s broader research and innovation agenda [16,64]. The AVAREF program is using an ecosystem and life-cycle approach to support end-to-end production from research and development, through clinical research, ethical and regulatory oversight, and manufacturing, to safety monitoring [16]. Through coordinated scientific advice meetings, early engagement with developers, and the development of regulatory guidance and toolkits, the forum has helped strengthen the regulatory environment for vaccine research and development conducted in Africa. These efforts contribute to shortening timelines for clinical trials, improving regulatory predictability, and fostering greater confidence among global and regional partners investing in vaccine development on the continent [16]. Looking ahead, the operationalization of the African Medicines Agency offers an opportunity to institutionalize many of the collaborative regulatory practices pioneered by AVAREF, further strengthening regulatory convergence, preparedness for public health emergencies, and equitable access to vaccines in the WHO African Region [64,65].

Notwithstanding these gains, AVAREF operates within a fragmented regulatory landscape characterized by heterogeneous legal frameworks, variable national regulatory authority capacity, and uneven adoption of joint review processes. These constraints can limit efficiency gains and contribute to persistent delays in product approval. The establishment of the African Medicines Agency represents a critical step towards continental harmonization, but its impact will depend on sustained political commitment, broader treaty ratification, and alignment with existing collaborative mechanisms.

## 9. Implementation Research and Data-Driven Program Transformation

Implementation research as a tool for data-driven program management is central to post-PHEIC immunization strengthening in the WHO African Region. While rapidly restoring coverage levels is crucial, sustained progress depends on the effectiveness of translating policies into operational delivery across diverse, often fragile contexts. Implementation research focuses on understanding and addressing the factors that affect the adoption, integration, and sustainability of health systems interventions in the real world [67]. When combined with systems thinking, stakeholders are better equipped to understand and navigate the inherent complexity of systems.

In the post-PHEIC period, there should be intensified action to mainstream implementation research into immunization programs across the region, especially in large countries with substantial accumulation of zero-dose and under-vaccinated children. This shift will require greater reliance on robust subnational immunization data systems and the institutionalization of data triangulation across multiple sources to guide adaptive learning within routine program cycles in specific contexts. Development of evidence-based implementation plans, their monitoring, and the use of data from implementation could go a long way toward supporting program transformation.

### 9.1. Immunization Implementation Research

There has been a stronger strategic focus on implementation research and systems thinking in the WHO African Region post-PHEIC to increase the use of theory-informed determinant frameworks to describe factors influencing vaccination efforts, illustrate the complexity of these factors by uncovering their interrelationships, and develop documentation frameworks to support cross-context learning [67].

At the regional level, the Consolidated Framework for Implementation Research has been employed in rapid reviews to investigate the factors influencing the second dose of measles vaccination, human papillomavirus vaccination, malaria vaccination, and polio SIAs in the region. In these studies, researchers also integrated causal loop diagramming to qualitatively depict a more holistic system map of the interconnections among determinants and feedback loops. Identifying feedback loops within the relationship between determinants can inform more precise interventions [67,68].

Within countries, there has been an emergence of various embedded implementation research activities in immunization programs [44,45]. All these efforts are yielding newer, practical, evidence-based implementation strategies for vaccination within PHC that can be useful even beyond where they are originally tested.

As novel strategies for enhancing vaccination efforts are demonstrated to be effective in specific settings, it is essential to disseminate them rapidly and with sufficient detail to enable cross-context learning, whether within or across countries. This motivated regional specialists to develop the Documenting and Reporting Implementation strategies of Vaccination Efforts (DRIVE) framework, an eight-component tool that leverages existing implementation research guidance for documenting strategies [68]. The aim of this documentation framework is to improve the systematic dissemination of implementation strategies employed in vaccination programs, thereby supporting learning and knowledge exchange.

### 9.2. Data Systems and Digital Health

The recovery period has also witnessed the expanded use of digital immunization platforms, including District Health Information Software 2 (DHIS2)-based modules, electronic immunization registries, and interactive dashboards that provide near-real-time visibility of surveillance and immunization coverage indicators [62,69]. These systems are increasingly central to program performance management, enabling monitoring of antigen-specific coverage, dropout rates, stock levels, and surveillance sensitivity indicators at both national and subnational levels.

By triangulating routine immunization data with surveillance information, campaign monitoring results, and demographic estimates, countries are better able to identify zero-dose and under-vaccinated populations. Such integrated analysis supports targeted microplanning, prioritization of underperforming districts, and more responsive supervisory action, particularly in settings affected by conflict, population displacement, or urban informal settlements.

Digital systems have also strengthened vaccine introductions and outbreak response [18,19,20,21,22]. Real-time dashboards enable tracking of rollout progress, identification of operational bottlenecks, and rapid course correction during rollout of new and under-utilized vaccines. Similarly, strengthened electronic surveillance platforms improve the timeliness of outbreak detection and laboratory confirmation for measles, yellow fever, polio, and other VPDs, reinforcing the link between surveillance and immunization performance.

However, digital transformation alone does not guarantee improved outcomes. Sustained impact requires investment in data quality assurance, workforce analytical capacity, interoperability between surveillance and immunization platforms, and structured feedback mechanisms that translate data into action. Embedding data triangulation and routine performance review processes within national and subnational program cycles will be essential for accelerating equity gains and advancing toward IA2030 targets [48].

### 9.3. Constraints and Course for Digital Transformation

In the WHO African Region, digital transformation is occurring within a set of structural and operational constraints that shape both implementation and impact. Despite widespread adoption of platforms such as DHIS2, many countries continue to face infrastructure limitations, including unreliable electricity, intermittent internet connectivity, and high data costs, particularly in rural and conflict-affected settings. These constraints necessitate offline-first system architectures and hybrid connectivity solutions but also contribute to delays in data transmission and incomplete reporting.

A second major challenge is fragmentation and limited interoperability across digital health ecosystems. Immunization information systems, surveillance platforms, logistics management tools, and civil registration systems are often developed in parallel, with weak integration and inconsistent data standards. Emerging initiatives (such as the push by the Africa Centers for Disease Control and Prevention [Africa CDC] toward sovereign, interoperable data architectures and federated data systems) highlight both the scale of this challenge and the strategic direction required to address it [76]. Practical use cases, including electronic birth notification systems linking health facilities to civil registration and vital statistics (as implemented in countries such as Namibia), illustrate how interoperability can strengthen life-course immunization tracking and population denominator accuracy.

Third, workforce capacity and data use culture remain uneven. While digital tools have expanded rapidly, the ability to analyze, interpret, and act on data at subnational levels often lags behind system deployment. This results in persistent gaps between data availability and programmatic decision-making, particularly in decentralized health systems.

Finally, data quality and governance challenges (including incomplete reporting, denominator inaccuracies due to weak population registries, and unclear data ownership frameworks) continue to limit the reliability and use of digital data for performance management. Country experiences such as Botswana’s investments in integrated digital health platforms demonstrate the potential for strengthening real-time data use but also underscore the need for sustained investments in governance, standards, and system integration [77].

Addressing these Africa-specific constraints will be critical to realizing the full potential of digital transformation. This requires coordinated investments in infrastructure, interoperability frameworks, workforce capacity, and data governance, aligned with continental initiatives on digital health and data sovereignty [77]. Without interoperability and consistent use of data at subnational levels, digital expansion risks reinforcing fragmentation rather than enabling precision delivery.

## 10. Immunization Financing and Sustainability

The cost structure and financing of national immunization programs in the WHO African Region have evolved substantially over the past decade. As vaccine portfolios have expanded and technical tools improved, domestic financing has stagnated or declined in many countries. In 2024, only 12 of 47 (25.5%) countries reported increases in immunization expenditure compared with the previous year, while 23 (48.9%) countries reported an average decrease of around 50% relative to prior-year spending [9]. Twenty-seven countries fund less than 50% of their vaccine requirement costs from domestic resources, and only a small number allocate more than 2% of PHC budgets to vaccines. These patterns underscore structural dependence on external financing and expose a critical vulnerability in sustaining immunization gains. The sustainability of immunization programs in the region is therefore increasingly determined not by vaccine availability, but by fiscal commitment and the predictability of operational funding.

Gavi provides substantial funding to immunization programs in many countries in the region [46]. However, four countries in the region (Congo, Côte d’Ivoire, Ghana, and São Tomé and Príncipe) are now in the Gavi accelerated transition phase, facing rising co-financing obligations. Three countries (Angola, Eswatini, and Cabo Verde) are within Gavi’s newly established Middle-Income Country framework, while eight countries (Algeria, Botswana, Equatorial Guinea, Gabon, Mauritius, Namibia, Seychelles, and South Africa) are fully self-financing or were never eligible for Gavi support. For countries in accelerated transition, sustaining costly vaccine introductions such as Men5CV or HPV multi-age cohort campaigns requires fiscal planning that extends beyond donor cycles. Without predictable domestic resource mobilization, progress risks reversal once external catalytic funding diminishes.

In general, countries eligible for Gavi support are expected to finance traditional childhood vaccines from domestic resources, and to co-finance newer vaccines, maintain cold chain expansion, fund data systems, conduct periodic SIAs, and sustain outbreak preparedness and response [46]. This has occurred amid broader macroeconomic constraints, debt pressures, and competing health priorities. This evolving cost structure is further compounded by emerging external financing shocks. Recent reductions in United States Government contributions to key multilateral health financing mechanisms, including Gavi and WHO, alongside broader stagnation or reprioritization of donor funding, have introduced new uncertainty into the global immunization financing landscape [47,78]. These shifts risk widening existing financing gaps at a time when program costs are increasing, and countries are transitioning toward greater domestic responsibility, thereby heightening concerns about the sustainability of immunization gains in the region.

The Immunization Agenda 2030 and the Addis Declaration on Immunization both emphasize that immunization is a core government responsibility, requiring stable budget lines, medium-term expenditure frameworks, and integration into PHC financing platforms. Sustainable financing is not simply the presence of funds, but the capacity to mobilize and use domestic and external resources efficiently to meet current and future immunization goals. In practice, this means aligning immunization with broader PHC and health security investments, embedding vaccines within national health insurance schemes where feasible, and strengthening public financial management systems to reduce delays in fund disbursement.

However, opportunities do exist. The renewed political visibility of health security following the COVID-19 pandemic, the economic framing of vaccination as a human capital investment, and integration with PHC reforms create entry points for stronger domestic commitment [78]. Nonetheless, the post-PHEIC period suggests that political recognition has not yet translated consistently into sustained fiscal prioritization [78,79]. Without deliberate action to stabilize and increase domestic financing, the region risks a cycle in which vaccine introductions expand while operational funding for delivery, supervision, and surveillance remains insufficient, undermining the very impact those vaccines are intended to achieve.

The next phase of immunization recovery in the WHO African Region must treat financing reform as central, not peripheral to system resilience. Predictable domestic financing, aligned with PHC reform and transition planning, is a precondition for sustaining disease control gains and advancing toward IA2030 targets.

## 11. Discussion

The post-PHEIC experience of 2023–2025 in the WHO African Region demonstrates that immunization recovery is not a linear return to pre-pandemic performance, but a non-linear system transition shaped by interacting structural constraints and policy responses. Importantly, the region appears to have reached a coverage plateau, where further gains require system transformation rather than incremental scale-up. Africa’s immunization challenge is no longer primarily one of access, but of delivery precision; reaching the right populations, in the right places, with the right strategies. Three core insights emerge. Firstly, recovery must be designed as a transformation, embedding flexibility, catch-up, and integration into routine practice. Secondly, outbreak prevention and response are inseparable from strong immunization systems and surveillance, requiring predictable financing and accountability. Thirdly, new vaccines can be catalysts for system strengthening when introduced deliberately, in a way that strengthens each of the immunization system components, with community engagement and PHC integration at the core. As the region looks beyond immediate recovery, the challenge is to consolidate these gains into durable progress toward IA2030. The post-PHEIC period has shown that even after profound disruption, immunization can regain momentum through steady leadership, partnership, and evidence-informed adaptation. The task now is to ensure that this momentum is accelerated, equitably financed, and translated into lasting protection against VPDs.

Despite the gains, equity gaps have persisted in the post-PHEIC period. Zero-dose and under-vaccinated children remain disproportionately concentrated in remote rural areas, informal urban settlements, conflict-affected regions, and other marginalized populations [80]. Equity-first approaches (particularly those targeting zero-dose children) must become the central metric of program performance. While many national recovery plans emphasize catch-up vaccination, implementation has often been constrained by limited domestic financing, competing health priorities, and weak subnational delivery capacity. The high levels of inequity underscore the potential of implementation research in vaccination programs in the WHO African Region [67]. Health systems are inherently complex adaptive systems, and the interplay of contextual factors can differ from one community to another, leading to disparities in vaccination coverage [70]. Therefore, to go beyond current coverage levels and ensure equitable progress across diverse contexts, immunization programs need to embed implementation research as a management practice at both national and subnational levels. This allows for routine investigation of granular context-specific barriers and facilitators of vaccination efforts and co-develop practical solutions to overcome barriers and maintain facilitators [67]. In addition, strengthened and harmonized regulatory systems facilitate clinical research and ultimately increase access to quality-assured vaccines that are safe and effective.

Mass vaccination campaigns have been a central component of post-PHEIC recovery efforts in the WHO African Region, particularly for measles-rubella elimination, considering the rapid accumulation of unvaccinated young cohorts of children, which poses a direct risk for outbreaks. However, heavy reliance on campaigns risks obscuring persistent weaknesses in routine immunization systems. While campaigns can rapidly boost population immunity and prevent outbreaks, it is critical for countries to make parallel investments in routine service delivery, supply chains, and community engagement to ensure durable control of vaccine-preventable diseases in the WHO African Region. This transition would reflect a broader shift from episodic campaign-driven approaches to continuous, integrated delivery platforms embedded within PHC systems.

Strong surveillance systems constitute a critical backbone of disease elimination and eradication efforts. High-quality case-based surveillance enables early detection of outbreaks, timely laboratory confirmation, and rapid implementation of response measures. In the WHO African Region, surveillance platforms initially strengthened through the polio eradication initiative have been increasingly used to support the monitoring of multiple vaccine-preventable diseases. With the winding down of the Global Polio Eradication Initiative and the declining donor resources for vaccine-preventable disease surveillance, it is high time that countries mobilize their own resources to sustain the gains and strengthen their integrated surveillance systems [47]. It is impossible to attain VPD elimination and eradication targets and monitor the impact of interventions without having clear visibility on the epidemiological trends. Therefore, continued investment in surveillance sensitivity, laboratory capacity, and real-time data analysis will be essential for identifying immunity gaps, guiding targeted immunization interventions, and verifying progress toward elimination and eradication goals. The Regional Investment Case for VPD Surveillance (2021–2030) outlines the key elements that countries can use to tailor and build upon their surveillance systems [81].

By preventing infections and reducing the need for antibiotic treatment, vaccines lower the selective pressure that drives antimicrobial resistance. Surveillance systems also provide an opportunity to generate evidence on how immunization contributes to mitigating antimicrobial resistance. Laboratory surveillance can track changes in resistance patterns in pathogens such as *Streptococcus pneumoniae* following vaccine introduction, while routine surveillance of vaccine-preventable diseases can indicate reductions in antibiotic use. Integrating immunization, laboratory, and antimicrobial use data through existing surveillance platforms can strengthen evidence for the vaccine-antimicrobial resistance linkage [82]. However, vaccines remain an underexplored tool for addressing antimicrobial resistance in Africa, highlighting the need for stronger integration of vaccination strategies within national antimicrobial resistance policies and surveillance frameworks [82].

An additional strategic opportunity emerging in the post-PHEIC period is the accelerated digital transformation of immunization and PHC systems across the WHO African Region. Digital platforms are increasingly enabling countries to track vaccination status, identify zero-dose and under-vaccinated populations, and guide targeted outreach strategies at subnational levels [18,19,20,21,22,62,69]. Evidence suggests that integrated digital health systems can improve program management, enhance service coordination, and support personalized communication with caregivers through reminders and alerts, thereby reducing missed opportunities for vaccination and improving equity in coverage [83]. Africa CDC has articulated a continental vision for PHC digitalization, emphasizing interoperable digital public infrastructure, data intelligence, and integrated surveillance as foundational elements for universal health coverage and health security [84]. The alignment of immunization data systems with broader PHC digital architectures offers an opportunity to strengthen data-driven decision-making, support real-time monitoring of vaccine introductions and outbreak response, and enhance the visibility of underserved populations. However, realizing these benefits will require sustained investment in interoperability standards, workforce analytical capacity, data governance, and integration of digital tools into routine program management rather than treating them as parallel technological initiatives. The digitalization of PHC and immunization systems represents a critical enabling strategy for accelerating progress toward IA2030 goals while strengthening health system resilience in the WHO African Region.

In general, uncertainty remains regarding the durability of post-PHEIC recovery gains in the WHO African Region. Coverage improvements may reflect short-term campaign effects rather than sustained routine performance. In addition, reliance on national-level data limits visibility into subnational dynamics, and discrepancies across data sources may obscure true program performance. These uncertainties underscore the need for strengthened data systems and real-time performance monitoring.

## 12. Conclusions

Regional coverage for most vaccine doses in the WHO African Region has returned to 2019 pre-pandemic levels, but restoration of pre-pandemic performance is not an adequate benchmark for success. This recovery masks a structural plateau in performance. Many countries in the region entered the COVID-19 pandemic with suboptimal routine immunization coverage, persistent inequities, and fragile surveillance systems. Returning to this baseline risks normalizing vulnerability and perpetuating cycles of outbreaks and reactive responses. Post-PHEIC recovery must therefore be framed as a transformational agenda, not a corrective one. Prioritizing equity through stronger routine immunization services, embedding immunization within primary health care, and treating surveillance as a core public good are essential to reducing vulnerability to recurrent outbreaks. At the same time, implementation research should guide context-specific integration and sustain performance in fragile settings. The post-PHEIC period offers a critical opportunity to position immunization as a cornerstone of Africa’s Health Security and Sovereignty Agenda. However, realizing this will require sustained political leadership, increased domestic financing, and tighter alignment of partner support with country priorities. Without these shifts, cycles of outbreaks and reactive responses will persist despite the availability of effective tools and strategies. Finally, achieving sustained progress will require a deliberate shift toward precision delivery, equity-first strategies, and system transformation aligned with the Immunization Agenda 2030.

## Data Availability

Data are contained within the article.

## Abbreviations

AFRO-MoVE	African Region Monitoring Vaccine Effectiveness
AVAREF	African Vaccine Regulatory Forum
COVID-19	Coronavirus disease 2019cVDPV: circulating vaccine-derived polio virus
DHIS2	District Health Information Software 2
DRIVE	Documenting and Reporting Implementation strategies of Vaccination Efforts
GPEI	Global Polio Eradication Initiative
HPV	Human papillomavirus
IA2030	Immunization Agenda 2030
ICC	Inter-Agency Coordinating Committee
IPV	Inactivated polio vaccine
MCV	Measles-containing vaccine
Men5CV	Pentavalent meningococcal ACWYX conjugate vaccine
MenACV	Meningococcal A conjugate vaccine
MNT	Maternal and neonatal tetanus
MOV	Missed opportunity for vaccination
NITAG	National Immunization Technical Advisory Group
PHC	Primary Health Care
PHEIC	Public Health Emergency of International Concern
PMVCs	Preventive mass vaccination campaigns
RCV	Rubella-containing vaccine
RITAG	Regional Immunization Technical Advisory Group
SIA	Supplementary immunization activity
VPD	Vaccine-preventable disease
WHO	World Health Organization
WUENIC	WHO–UNICEF Estimates of National Immunization Coverage
